# A Case of Urethral Diverticulum with Surgical Repair Using Cadaveric Pericardial Tissue

**DOI:** 10.1155/2018/6183618

**Published:** 2018-11-04

**Authors:** Loren Custer, Morris Jessop, Stanley Zaslau, Robert Shapiro

**Affiliations:** West Virginia University, Department of OB/GYN, Morgantown, WV, USA

## Abstract

A urethral diverticulum is a relatively uncommon finding. The estimated prevalence is approximately 1-5% in the general population. While the definitive treatment is surgical correction, there are limited studies guiding the best approach to repair. This is the case of a 48-year-old female who initially presented with vaginal discharge, dysuria, and dyspareunia. MRI revealed the diagnosis of suspected urethral diverticulum. The patient was treated with surgical correction with the aid of needle localization prior to the procedure. After the diverticulum was excised, the resulting defect in the urethra was successfully closed with cadaveric pericardial tissue. A urethral diverticulum should be considered in the differential diagnosis when a patient presents with symptoms such as recurrent urinary tract infections (UTIs) vaginal mass, dysuria, dyspareunia, or vaginal discharge. The use of cadaveric tissue augments the surgical technique for repair.

## 1. Introduction

A urethral diverticulum is defined as an abnormal outpouching of the urethral mucosa into the adjacent tissue. As noted above, the prevalence is approximately 1-5% in the general population [1] and the incidence is estimated to be less than 0.02 percent per year [2]. This condition is found more commonly in women than men and usually occurs between 20 and 60 years old [3]. There are several theories to explain the cause of these diverticula. One proposed theory is that of chronic infection/inflammation of the periurethral glands resulting in abscess formation that can communicate with the urethral lumen [3]. Another proposed theory is that trauma via either surgical procedures (ex sling procedures), physical injury, or child birth may cause formation of urethral diverticula later in life [4–6]. Other known risk factors include African American race [1] and female gender [3].

The classic triad of symptoms reported in the literature is postvoid dribble, dyspareunia, and dysuria [7]. Other common presenting symptoms include recurrent urinary tract infections, urethral stones, and tender vaginal mass [7, 8]. The current standard for diagnosis is by MRI [9–11]. There have also been studies utilizing transvaginal ultrasound [12]. MRI, however, can give additional information such as if a mass were present within the diverticula [10].

Treatment is indicated if the patient is experiencing bothersome symptoms or if malignancy is suspected. Transvaginal diverticulectomy and urethral repair is the current procedure of choice. The reported success rate is approximately 70-86% [13]. There are several known postoperative complications reported in the literature including urethral stricture, ureterovaginal fistula, stress incontinence, and recurrent UTIs [14]. The risk of complications increases with repeat procedures [15].

## 2. Case Presentation

**Figure 1:**
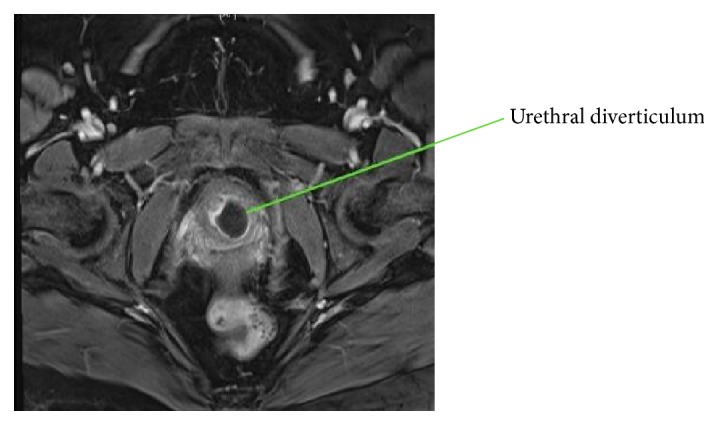
Pelvic MRI, axial view of urethral diverticulum.

**Figure 2:**
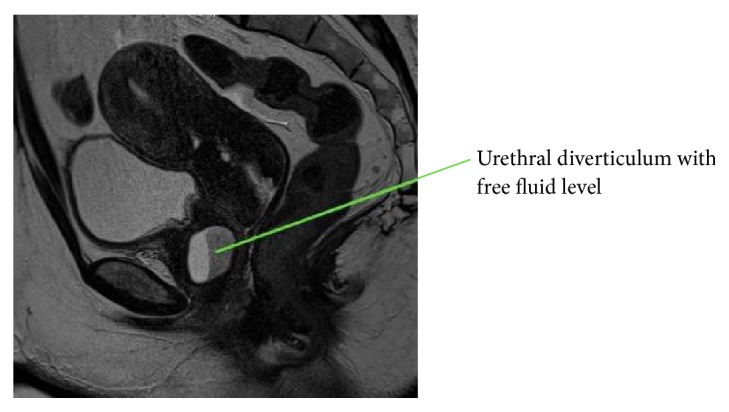
Pelvic MRI, sagittal view of urethral diverticulum.

This is the case of a 48-year-old G2P2002 who originally presented to our office in March 2016 with vaginal discharge, dysuria, and dyspareunia. She had previously been evaluated by her primary care physician (PCP) where a small cyst on the anterior vaginal wall was drained. She had received antibiotic treatment without relief of her symptoms. Her past medical history was significant for multiple sclerosis, Crohn's disease, and anxiety/depression. Her surgical history was significant for cesarean section × 2, bilateral tubal ligation, polypropylene midurethral sling procedure, and cholecystectomy. On physical examination, she was noted to have a small anterior vaginal wall fold near the urethra. MRI showed a cystic mass posterolateral to the urethra measuring 2.1 × 1.7 × 2.3 cm likely representing a urethral diverticulum. The mass had signs of infection/inflammation (Figures 1 and 2). Of note, the patient also had a long history of abnormal uterine bleeding and was found to have fibroids on MRI. She underwent hysterectomy with concurrent repair of the urethral diverticulum.

In June 2016, she underwent total abdominal hysterectomy, bilateral salpingectomy, and adhesiolysis. After the hysterectomy was complete, excision of her previous midurethral sling was also performed due to the patient's chronic groin pain and recurrent UTIs. Approximately 2 cm of the polypropylene mesh was freed from the periurethral space and partially excised to the inferior pubic rami bilaterally. At the time of excision, no diverticulum was able to be identified and only an area of inflammation was visualized. A 20 gauge spinal needle was passed through that area in an attempt to aspirate fluid, but no fluid was able to be retrieved. Cystoscopy was also performed and no ostia or communication suggestive of a diverticulum was visualized. Postoperatively, patient had no complications. Although the diverticulum was not able to be isolated and repaired, the patient initially had improvement of her symptoms.

In January 2017, patient returned with vaginal discharge, status post “vaginal cyst drainage” again with her PCP, along with antibiotic therapy. MRI was repeated and significant for persistence of the cystic mass/diverticulum noted on prior MRI. Her symptoms however improved after antibiotic therapy. Further treatment was subsequently delayed due to left breast discharge and subsequent diagnosis of a breast mass.

In October 2017, patient returned with 2-week history of dysuria along with persistent discharge and urinary dribbling. MRI was repeated and significant for slight increase in size of the urethral diverticulum. The case was discussed with our staff in interventional radiology. They offered needle localization of the diverticulum prior to attempting repeat excision.

**Figure 3:**
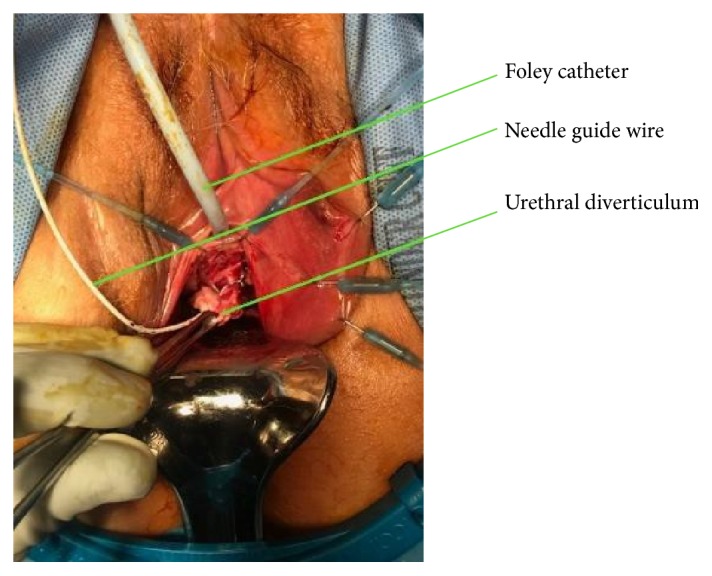
Urethral diverticulum isolated with needle localization.

**Figure 4:**
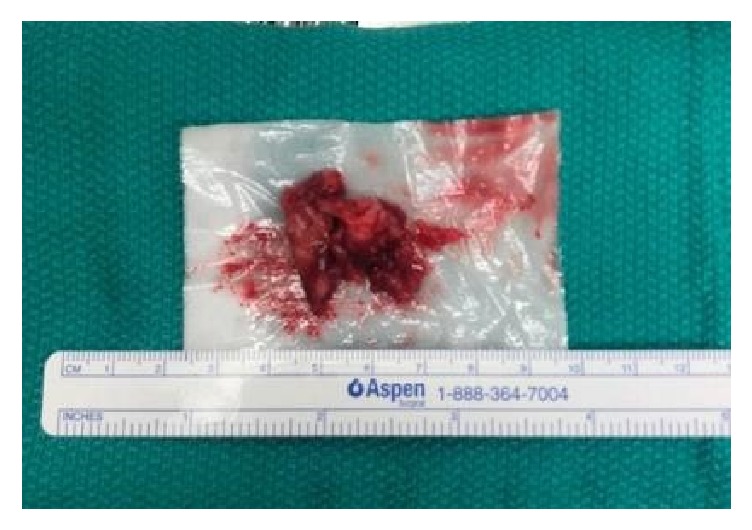
Urethral diverticulum, after removal.

In January 2018, the patient underwent the following procedure: transvaginal ultrasound was used to identify a “thick walled diverticulum” and a 5 French catheter needle was placed into this cavity. A small wire was then passed through the catheter and into the diverticulum for localization. The catheter was removed and the wire was then secured to the patient's thigh. A u-shaped incision was then made, proximal to the urethra. After the vaginal epithelium was dissected away, the diverticulum was easily identified using the guide wire (Figure 3). The diverticulum was incised longitudinally and purulent material was subsequently drained. The diverticulum was dissected laterally and anteriorly away from the urethra and subsequently removed.  Figure 4 illustrates the size of the diverticulum. After the diverticulum was removed, there was an approximately 1 cm defect in the urethra due to a previous communication between the two areas. This was repaired with 3-0 vicryl; however, due to the chronic inflammation, the tissue was weak and a leak was noted after repair, evident after methylene blue dye injection. Therefore, the defect was reopened and reapproximated with 3-0 vicryl. This time, cadaveric pericardium was anchored over the first layer of repair. The cadaveric pericardium was soaked in normal saline for rehydration per package insert instructions. The vaginal epithelium was also mobilized and placed over the cadaveric tissue for a 3rd layer of repair to ensure adequate closure. The U-shaped incision was then closed and Premarin cream was placed over the suture line. A new foley was also placed at the end of the case. Of note, culture from the fluid expressed from the diverticulum at time of repair was negative for growth.

At her 5-week postop visit, a fluorocystogram was performed and was normal. The foley catheter was subsequently removed. At 3 and 6 months after surgery, the patient was completely asymptomatic and denied leakage of urine, urinary incontinence, dysuria, or discharge.

## 3. Discussion

Given the rare occurrence of urethral diverticula, there is often a delay in detection leading to persistent symptoms, such as postvoid dribble, dyspareunia, or vaginal discharge, especially if the patient had prior pelvic surgery. [16]. A delay in diagnosis occurred with our patient as well. She had received several courses of antibiotic treatment and localized aspiration prior to presenting to our clinic. This may have contributed to the inability to find the urethral diverticulum during the first surgery. The lack of an acute infection within the diverticulum likely made it too small to recognize. Isolation of the diverticulum during surgical exploration becomes easier if a vaginal mass (usually along the anterior vaginal wall) is palpable or visible.

The cause of this patient's urethral diverticulum is difficult to ascertain. It is notable that our patient had a history of a prior polypropylene midurethral sling procedure. The authors speculate increased intraurethral pressure resulting from the placement of the polypropylene sling could have caused the diverticulum to form. This is not a novel theory as other case reports have been published on urethral diverticula associated with prior urethral sling surgery [17]. However, no clear evidence exists citing urethral diverticulum as a complication of sling surgery. Moreover, it is difficult to exclude the possibility the patient might have had a very small diverticulum before the sling operation which got worse as a result of surgery.

MRI is currently the preferred imaging modality to diagnose urethral diverticula [9–11], although studies are limited to guide which type of imaging is best. MRI is useful because it can better differentiate between solid masses and diverticula. MRI can also better delineate the extent of the diverticulum, aiding in preoperative planning for repair [18]. In our case, MRI was useful in determining diagnosis; however, due to the location it was still difficult to access during the patient's first surgery. Alternative methods for diagnosis include ultrasound and CT scan. While ultrasound has its advantages, such as limited cost and ease of use, the limitation is lower quality images. In our case, the diagnosis was made via MRI; however, the use of real-time ultrasound imaging was helpful in the operating room for localization during repair. This should be considered in future diverticular repairs.

The technique described in the literature for closing a urethral defect involves a multilayer closure. The urethral mucosa itself is first reapproximated over a foley catheter. The next layer of closure is the periurethral fibromuscular, followed by the anterior vaginal wall incision. In our case, the urethral tissue as well as the tissue around the urethra was weak and inflamed from chronic irritation and infection. To correct for weak tissue, cadaveric pericardial tissue was sutured over the urethral closure to reinforce the area. There have been similar techniques reported in the literature with good outcomes [19–22]. We found one case similar to ours where repair was successfully performed with bovine pericardium [19]. Upon literature review, there have been small studies evaluating success rates between primary anastomosis as described above versus closure using substitution urethroplasty (i.e., graft tissue). One study performed by Alphs et al. noted similar outcomes between the two techniques [20]. Finally, we applied estrogen cream to the wound in the immediate postoperative setting to promote healing of the vaginal epithelium [21].

Upon further investigation of urethral reconstruction for other reasons (such as hypospadias), other materials have been used with success, such as buccal mucosa [22], bladder mucosa [23], and colonic mucosa [24]. Therefore, substitution urethroplasty has been shownto have good outcomes as illustrated in our case and should be considered when native tissue is not well suited for repair.

Another consideration in this case would have been to perform a Martius modified labial fat pad flap (MMLFPF), commonly known as the Martius flap. The Martius flap has been described in the literature most commonly for vaginal fistula repair [25]. This technique has been noted to be successful in cases where vaginal tissue integrity is a concern [26]. We chose cadaveric pericardial tissue over a MMLFPF to diminish risk of potential long term patient sequelae such as labial pain, numbness, and distortion, all of which have been described in the literature [27].

## 4. Conclusions

1. Urethral diverticulum should be in the differential diagnosis when a patient presents with recurrent urinary symptoms, a vaginal mass or incontinence, especially if they have a history of periurethral procedures/trauma.
2. Needle localization of urethral diverticulum at time of repair is a strategic approach to adequately and efficiently repair the defect.
3. The use of cadaveric pericardium is an excellent technique when repairing defects in the urethral mucosa if the surrounding tissue is too weak for repair.
